# P-989. Respiratory virus infections and symptoms for mothers of infants with acute respiratory infection

**DOI:** 10.1093/ofid/ofae631.1179

**Published:** 2025-01-29

**Authors:** Anne-Marie Rick, Monika Johnson, Sara Walters, Judith M Martin, John V Williams

**Affiliations:** University of Pittsburgh, Pittsburgh, Pennsylvania; University of Pittsburgh, Pittsburgh, Pennsylvania; University of Pittsburgh, Pittsburgh, Pennsylvania; University of Pittsburgh, Pittsburgh, Pennsylvania; University of Pittsburgh, Pittsburgh, Pennsylvania

## Abstract

**Background:**

It is recommended that women receive 4 vaccines (influenza, SARS-CoV-2, respiratory syncytial virus (RSV), Tdap) during pregnancy. Among infants with acute respiratory infection (ARI), limited data exist on the burden of viral infections in their mothers. The objective of this study was to describe ARI symptoms and viral infections in the biologic mothers of infants with ARI.

**Figure:**
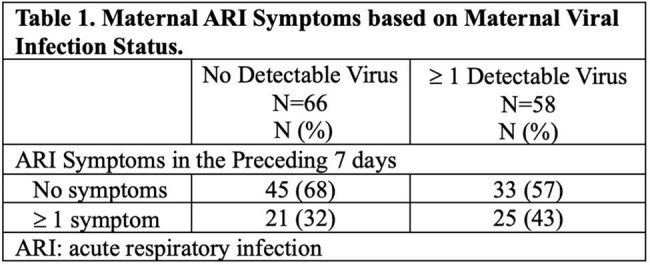

**Methods:**

Symptomatic infants < 12 months of age with ≥1 ARI symptom for < 7 days enrolled in the New Vaccine Surveillance Network (NVSN) in Pittsburgh, Pennsylvania were recruited along with their biologic mothers to a sub-study during 3 consecutive influenza seasons from 2021 to 2024. Infant ARI symptoms and mid-turbinate (MT) nasal swabs were collected through NVSN. For mothers, we collected presence/absence of ARI symptoms in the preceding 7 days and MT nasal swabs. All MT swabs were tested with the same in-house RT-PCR for 18 viruses/subtypes. Cycle threshold (CT) was compared between mothers and infants using Student’s T-test, where higher CT indicates lower viral load.

**Figure 1.**
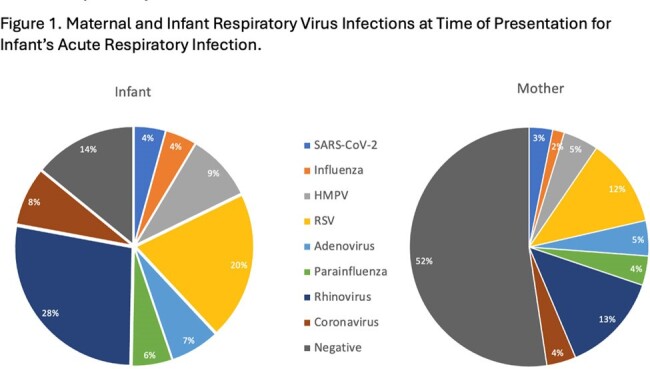
Maternal and Infant Respiratory Virus Infections at Time of Presentation for Infant’s Acute Respiratory Infection.

**Results:**

From 124 mother-infant pairs, 100% (N=124) of infants vs. 37% (N=46) of women reported ≥ 1 ARI symptom in the preceding 7 days. 53% (N=66) of women had no detectable virus (Fig 1). 68% (N=45) of those women had no symptoms, while 32% (N=21) had ≥ 1 symptom (Table 1). From the 47% (N=58) of women with detectable virus, 45% (N=56) had one virus, 2% (N=2) had two (Fig 1). 97% (56/58) had the same virus as their infant. Vaccine preventable infections occurred in 17% of women (SARS-CoV-2 N=4, influenza N=2, RSV N=15; 20/21 were unvaccinated for that pathogen) and 39% of infants (SARS-CoV-2 N=6, influenza N=5, RSV N=25). CT for RSV and coronaviruses was higher (i.e. lower viral load) for women compared to infants (Fig 2). Among women with detectable virus, 57% (N=33) had no symptoms, while 43% (N=25) had symptoms (Table 1).

**Figure 2.**
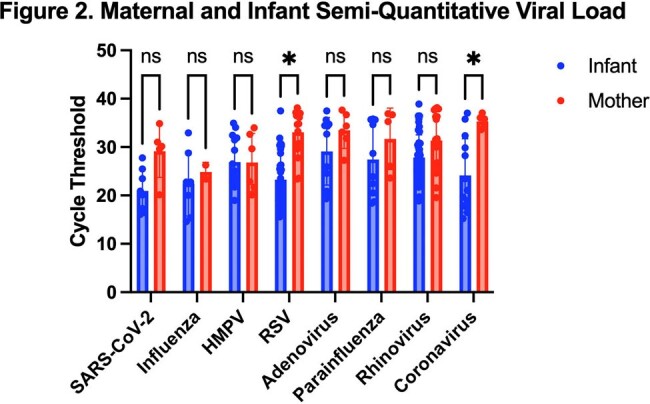
Maternal and Infant Semi-Quantitative Viral Load

**Conclusion:**

Concordant viral infection occurs in half of mothers who have an infant with ARI. However, semi-quantitative viral load is similar or lower in mothers, possibly due to prior infection or vaccine. Additionally, the majority of maternal infections are asymptomatic. Pregnancy vaccines are likely to further reduce detectable and symptomatic vaccine-preventable infections in mothers, which may further enhance infant protection.

**Disclosures:**

**Anne-Marie Rick, MD, MPH, PhD**, Pfizer: Advisor/Consultant **Judith M. Martin, MD**, Centers for Disease Control and Prevention: funding to the institution to support this work|Centers for Disease Control and Prevention: funding to the institution for unrelated work|Merck, Sharp and Dhome: funding to the institution to support this work|Moderna: funding to the institution for unrelated work|NIH: funding to the institution for unrelated work

